# Immunomodulatory components of *Trichinella spiralis* excretory-secretory products with lactose-binding specificity

**DOI:** 10.17179/excli2022-4954

**Published:** 2022-06-03

**Authors:** Natasa Ilic, Zanka Bojic-Trbojevic, Britta Lundström-Stadelmann, Danica Cujic, Ivana Mitic, Alisa Gruden-Movsesijan

**Affiliations:** 1 University of Belgrade, Institute for the Application of Nuclear Energy, Banatska 31b, 11080 Belgrade, Serbia; 2 Institute of Parasitology, Department of Infectious Diseases and Pathobiology, Vetsuisse Faculty, University of Bern, Länggassstrasse 122, 3012 Bern, Switzerland

**Keywords:** Trichinella spiralis, immunomodulation, galectin-1, proteomics

## Abstract

The immunomodulatory potential of *Trichinella spiralis* muscle larvae excretory-secretory products (ES L1) has been well documented *in vitro *on dendritic cells (DCs) and in animal models of autoimmune diseases. ES L1 products possess the potential to induce tolerogenic DCs and consequently trigger regulatory mechanisms that maintain immune homeostasis. The use of ES L1 as a potential treatment for various inflammatory disorders proved to be beneficial in animal models, although the precise immunomodulatory factors have not yet been identified. This study aimed at the isolation and characterization of ES L1 components that possess galectin family member properties. Galectin-1-like proteins (TsGal-1-like) were isolated from ES L1 based on the assumption of the existence of a lactose-specific carbohydrate-recognition domain and were recognized by anti-galectin-1 antibodies in Western blot. This TsGal-1-like isolate, similar to galectin-1, induced DCs with tolerogenic properties and hence, the capacity to polarize T cell response towards a regulatory type. This was reflected by a significantly increased percentage of CD4^+^CD25^+^Foxp3^+^ regulatory T cells and significantly increased expression of IL-10 and TGF-β within this cell population. Proteomic analysis of TsGal-1-like isolate by mass spectrometry identified nineteen proteins, seven with annotated function after blast analysis against a database for *T. spiralis* and the UniProt database. To our surprise, none of the identified proteins possesses homology with known galectin family members. Nevertheless, the isolated components of ES L1 possess certain galectin-1 properties, such as specific lactose binding and the potential to elicit a regulatory immune response, so it would be worth further investigating the structure of sugar binding within isolated proteins and its biological significance.

## Introduction

*Trichinella spiralis* is a parasitic nematode which spends its entire life cycle in a single host. The parasite enters the host organism through consumption of raw or undercooked meat containing *T. spiralis* infective larvae. These larvae are liberated in the stomach of the host with the aid of gastric juice, they migrate to the intestine where they invade intestinal epithelial cells, molt and develop to adult worms capable of reproduction. Through blood circulation, released newborn larvae reach their target site in muscle tissue and mature to muscle larvae (L1 larvae), while transforming the muscle cells into nurse cells that are surrounded by a collagen capsule (Despommier, 1998[8], Wu et al., 2008[61]). This capsule is a completely new entity for the host organism and it protects the larvae from the host defense mechanisms. From this immune privileged place, muscle larvae communicate with the host organism through their excretory-secretory products (ES L1), a complex mixture of glycoproteins with different functions (Nagano et al., 2009[40]). ES L1 proteins enable the parasite to invade the host organism, establish parasitism and ensure its survival by manipulating the host immune system. The chronic muscle phase of the infection is characterized by a dominance of a regulatory immune response that prevents excessive immune reactions aimed not only towards parasite antigens, but also bystander antigens, such as autoantigens and allergens (Sofronic-Milosavljevic et al., 2015[50]). We have shown that ES L1 products induce a tolerogenic phenotype in rat (Gruden-Movsesijan et al., 2011[17]) and human dendritic cells (DCs) (Ilic et al., 2018[27]), key antigen presenting cells responsible for the induction and maintenance of the host immune response. ES L1 products provoke Th2 and regulatory responses when applied *in vivo* in Dark Agouti rats, and their application prior to disease induction could ameliorate experimental autoimmune encephalomyelitis, an animal model of multiple sclerosis (Radovic et al., 2015[46]). Parasite-driven protection was associated with the induction of the regulatory cytokines IL-10 and TGF-β, and increased presence of CD4^+^CD25^+^Foxp3^+^ T regulatory cells (Treg), which could restore self-tolerance otherwise lost in autoimmune diseases (Maizels, 2016[38]). Several studies in the past aimed to identify components of *T. spiralis* ES L1 that possess immunomodulatory capacity and could therefore be used as therapeutics against chronic inflammatory diseases (Wei et al., 2021[60]; Kobpornchai et al., 2020[32]; Cvetkovic et al., 2016[7]; Du et al., 2011[11]), but they were not successful. 

Among parasite-derived molecules that may have an immunomodulatory potential are parasitic galectins, which could act as immunological mediators of homeostasis, similar to members of a galectin family originating from the vertebrates (Rabinovich and Ilarregui, 2009[45]; Young and Meeusen, 2002[66]). Galectins are a broad family of evolutionary conserved carbohydrate-binding proteins, present in both vertebrates and invertebrates, which are engaged in diverse physiological and pathophysiological activities (Camby et al., 2006[4]). Members of this family may not be particularly alike, but they all share a highly conserved carbohydrate recognition domain (CRD) that contains a ligand-binding pocket (or groove) with particular amino acids crucial for β-galactoside binding (Hirabayashi and Kasai, 1993[23]; Barondes et al., 1994[2]). Human galectin-1 was the first member of the family that was discovered (Barondes et al., 1994[2]; Cho and Cummings, 1995[5]) and since then various intracellular and extracellular activities of this lectin were revealed (Di Lella et al., 2011[9]). Galectin-1 participates in extracellular matrix (ECM) assembly and remodeling, regulation of cell proliferation, growth and migration, tissue development and differentiation, and plays an important role in fine tuning of innate and adaptive immune responses (Camby et al., 2006[4]). Its major role in immunoregulation is to suppress excessive inflammation and contribute to immune homeostasis (Ilarregui and Rabinovich, 2010[26]). Considering the functions galectin-1 performs in vertebrates, we here followed the hypothesis that candidates for immunomodulators among ES L1 products might be galectins or galectin-like molecules with similar function. 

The first galectin ever found in nematodes was the LEC-1 from *Caenorhabditis elegans*, with approximately 30 % amino acid sequence similarity to vertebrate galectins (Hirabayashi et al., 1992[24]). Eight amino acid residues (HxNxR…VxN…WxxExR) that are crucial for the β-galactoside binding site in vertebrate galectins (Hirabayashi and Kasai, 1994[22]) are present in invertebrate homologues, but in many cases some of these galactose-binding residues are missing, which could alter their ligand specificity (Dodd and Drickamer, 2001[10]). Over the years, the presence of galectins was revealed in a number of nematodes, such as *Haemonchus contortus* (Greenhalgh et al., 2000[16]; Hewitson et al., 2009[21]), *Toxocaris leonine* (Kim et al., 2010[30]), *Dirofilaria immitis* (Pou-Barreto et al., 2008[43]), *Wuchereria bancrofti* (Yasin et al., 2020[65]), and *Brugia malayi* (Hertz et al., 2020[20]). Nematode galectins, as also other parasite galectins, are induced during infection and participate in immune response modulation. Galectin from *H. contortus* changes cytokine production form T cells and monocytes (Hewitson et al., 2009[21]), while galectin from *T. leonine* induces IL-10 and TGF-β cytokine production, which contribute to the reduction of intestinal inflammation during dextran sulfate sodium induced inflammatory bowel disease (Kim et al., 2010[30]). According to the results available so far, only one galectin from the surface of *T. spiralis* muscle larvae (Tsgal, GenBank accession No. EFV62290) has been identified by immunoproteomics, and it is implicated in the invasion of host intestinal epithelial cells (Xu et al., 2018[63]). 

The aim of this study was to identify galectin-like proteins among ES L1 products of the *T. spiralis* muscle larvae based on their carbohydrate-binding specificity, since some of the ES L1 components were cross-reactive with antibodies raised against human and mouse galectin-1. The isolated ES L1 components (designated as TsGal-1-like components) possess lectin-like activity and induce tolerogenic DCs and CD4^+^CD25^+^Foxp3^+^ regulatory T cells *in vitro*. Mass spectrometry analysis of TsGal-1-like isolate identified proteins with different annotated functions, but neither possessed the sequence similar to human or mouse galectin-1.

## Materials and Methods

### Animals

Adult male Wistar rats for maintenance of *T. spiralis* strain ISS 7564 were obtained from Military Medical Academy (MMA, Belgrade, Serbia) and were housed under standard conditions (stable temperature 22-24 °C, humidity 45-60 %, 12:12 h light/dark cycle) of the animal facility with access to food and water ad libitum. Wistar rats, 2-4 months old, were infected by gastric inoculation with 8500 *T. spiralis* muscle larvae (L1 larvae). *T. spiralis* infective larvae were applied in 1 mL of PBS by gastric gavage, using flexible feeding tube that ends in a soft rounded bulbous tip that gently delivers the material through mouth to stomach. All procedures involving animals were approved and carried out in accordance with the guidelines and regulations by the local ethics committee of the Institute for the Application of Nuclear Energy-INEP, University of Belgrade, Serbia, Belgrade (permission date: February 23, 2017, in Belgrade, No. 04-406/5).

### Preparation of T. spiralis muscle larvae excretory-secretory products 

*T. spiralis* muscle larvae (L1 larvae) were recovered from Wistar rats 2 months p.i. by digesting the carcasses in pre-warmed gastric juice (1 % pepsin in 1 % HCl, pH 1.6-1.8) (Gamble et al., 2000[13]). Muscle larvae were kept under controlled conditions (37 °C, 5 % CO_2_) in complete Dulbecco's modified Eagle medium (DMEM) (Sigma-Aldrich, St. Louis, MO, USA), supplemented with 10 mM HEPES, 2 mM L-glutamine, 1 mM sodium pyruvate and 50 U/ml of Penicillin-Streptomycin (all from Sigma-Aldrich) for 18 h. ES L1 products were purified from culture supernatants according to the procedure described in Ilic et al. (2011[29]) and kept at -20 °C for further usage. 

### Immunohistological analysis

Live *T. spiralis* muscle larvae were fixed in cold ethanol and xylene and embedded in paraffin for histological analysis. Five-micron sections were deparaffinized in xylene, rehydrated with a graded alcohol series, washed and incubated with rabbit anti-human galectin-1 affinity-purified polyclonal antibody (1:1000) (Institute for the Application of Nuclear Energy - INEP, Belgrade, Serbia), overnight at 4 °C. After washing, sections were incubated with FITC-conjugated anti-rabbit IgG secondary antibody (1:1500) (Molecular probes, Thermo Fisher scientific, Waltham, MA, USA) or with biotin-labeled goat anti-rabbit IgG secondary antibody (1:500) (Vector Laboratories, Burlingame, CA, USA), for 1 h at room temperature (RT). Non-specific binding was assessed by omitting the anti-human galectin-1 incubation step. Indirect immunofluorescence (IIF) staining was examined by ultraviolet microscopy (AXIO Imager A1, Carl Zeiss AG, Goettingen, Germany). Incubation with biotin-labeled goat anti-rabbit IgG was followed by ABC (avidin/biotinylated horseradish peroxidase complex, Vector) (1:1000) for 30 min, and the reaction was developed by adding the chromogenic substrate. The stained sections were viewed using light microscopy (AXIO Imager A1, Carl Zeiss AG).

### SDS-PAGE, silver staining and Western blotting

Proteins were resolved by 10 % polyacrylamide gel electrophoresis (PAGE) under reducing conditions and visualized by silver staining, performed using the Silver Stain Kit (BioRad, Hercules, CA, USA) according to the manufacturer's instructions. Proteins separated on 10 % polyacrylamide gel were transferred to nitrocellulose membrane by Western blotting. Non-specific binding was blocked by TBS (50 mM Tris-HCl, 150 mM NaCl) pH 7.6/1 % BSA for 1 h at room temperature. The membranes were incubated overnight at 4 °C with rabbit anti-human galectin-1 (1:3000, INEP) and goat anti-mouse galectin-1 affinity-purified polyclonal antibody (1:1000, R&D, Minneapolis, MN, USA), followed by secondary antibody-HRP (biotin-labeled goat anti-rabbit IgG, Vector Laboratories, 1:3000; Rabbit anti-Goat IgG (H+L), HRP, Invitrogen, Waltham, MA, USA, 1:10000). Non-specific binding was assessed by incubation with secondary antibody only. Protein bands were detected by ABC (in case of biotinylated secondary antibody) or by adding 0.05 % 3.3'-diaminobenzidine tetrahydrochloride (DAB) (Vector Laboratories).

### Lactose affinity purification

Lactose-Sepharose 4B was prepared according to the slightly modified procedure described by Levi and Teichberg (1981[34]). Beads were equilibrated in 0.05 M phosphate buffered saline (PBS) containing 4 mM 2-mercaptoethanol and 2 mM EDTA (MePBS), 7.2. ES L1 products, 1 mg/ml, in a total volume of 3 ml of MePBS, were added to lactose-Sepharose 4B and incubated overnight at 4 °C, with constant shaking. Beads were extensively washed with MePBS to remove unbound proteins, and bound fractions were specifically eluted with 0.2 M lactose/MePBS. Remaining non-specifically bound proteins were eluted with Glycine-HCl pH 2.5. Eluted fractions (designated as TsGal-1 like) were dialyzed and concentrated using Amicon concentration unit with 10 kDa cutoff membrane. Samples intended for use in cell culture were filtered through 0.22 µM filters and kept at -20 °C until use.

### Binding assay

The lectin binding capacity of isolated TsGal-1-like proteins was tested in the solid phase assay with Matrigel (250 ng/well; BD Bioscences, Bedford, USA) immobilized on the surface of 96-well plates. Matrigel resembles the complex extracellular environment built-up from glycoproteins and proteins of extracellular matrix like collagen type I and IV, laminin, fibronectin, etc. The assay was performed with serial dilutions of TsGal-1-like proteins in MePBS (under reducing conditions) or in PBS (0-20 μg/ml) in the absence or presence of 0.2M lactose as an inhibitor of specific binding. TsGal-1 dilutions were added in triplicates, and incubated overnight, at 4 °C, followed by incubation with anti-human galectin-1 antibodies (1:500), 2 h, with shaking. For the detection of bound primary Ab, secondary biotin-labeled anti-rabbit IgG was used (1:3000, 30 min), followed by ABC (1:1000) for 30 min and further with substrate and chromogen. The reaction was stopped with 0.2 M H_2_SO_4_ and optical densities were measured at 450 nm on an ELISA reader (VICTOR 1420 Multilabel Counter, Perkin Elmer, Waltham, MA, USA).

### Isolation and culture of immune cells

Monocytes and T cells were isolated from the blood of healthy donors, obtained after they gave written informed consent in accordance with the Declaration of Helsinki. This study was carried out in accordance with the recommendations of local Ethics Committee of INEP.

Peripheral blood mononuclear cells (PBMCs) from healthy donors were isolated by a density gradient centrifugation on Lymphoprep™ Density Gradient Medium (Stemcell Technologies, Seattle, WA, USA). The monocytes and CD3^+^T lymphocytes were purified from obtained PBMCs by using the Classical Monocyte Isolation Kit and the Pan T cell isolation kit (both from Myltenil Biotec, GmbH, Cologne, Germany) according to the manufacturer's instructions. The purity of CD14+ monocytes and CD3+ T cell populations was higher than 90 %.

The monocytes were cultivated in CellGro DC medium (Cell Genix CellGenix GmbH, Freiburg, Germany), supplemented with human recombinant GM-CSF (100 ng/ml) and IL-4 (20 ng/ml) (both from R&D Systems, Minneapolis, MN, USA), in a 24-well plate (0.5x10^6^ cells per well) for 6 days in CellGro DC medium, and the medium was refreshed on day 4. The impact of ES L1 and TsGal-1-like proteins on the maturation of DCs was determined by adding ES L1 (50 µg/ml) and TsGal-1-like proteins (5 µg/ml) to the culture of immature DCs on Day 4, for 48 h. The specificity of TsGal-1-like proteins interaction with cell surface glycoproteins was assessed by inhibition of binding with lactose. TsGal-1-like proteins were incubated with an equal volume of 0.2 M lactose (TsGal-1-like/Lac) in CellGro DC medium supplemented with 4 mM 2-mercaptoethanol, overnight, at 4 °C, with shaking and then added to the culture of immature DCs on Day 4. To induce mature DCs, the cells were stimulated with LPS from *Escherichia coli* (200 ng/ml, Sigma-Aldrich) and human recombinant IFN-γ (50 ng/ml, R&D Systems) on Day 5, for the next 24 h. The cells were harvested and prepared for phenotypic analyses by flow cytometry or for functional assays with T cells. 

### Allogenic T cell-stimulatory capacity of DCs

The capacity of treated and non-treated DCs to induce T cell polarization was assessed in an allogenic stimulation assay. Naïve allogenic T cells (1×10^5^/well) were co-cultivated with DCs (0.5x10^4^/well) in 96-well round-bottom plate in a final volume of 200 μl, for 6 days. For the flow cytometric detection of intracellular cytokines, the co-cultures were treated with phorbol myristate acetate (PMA) (20 ng/ml), inomycin (500 ng/ml) and monensin (3 µM) (all from Sigma-Aldrich) for the last 3 h of incubation. Cells were analyzed for the production of cytokines (IL-4, IL-10 and IFN-γ) by flow cytometry analysis (BD FACS LSR II flow cytometer). 

To analyze the capacity of stimulated DCs to induce regulatory T cells (Tregs), allogenic naïve T cells were primed with DCs at a 1:50 DC/T cell ratio for 5 days. Allogenic T cells (1×10^5^/well) were plated with DCs (0.2x10^4^/well) in 96-well round-bottom plate, and after 3 days of incubation, 20 IU⁄mL of human recombinant IL-2 (2 ng/ml, R&D Systems) were added. The culture was incubated the next 48 hours and for the last 4 h of incubation, the co-cultures were treated with PMA/ionomycin and monensin. The expression of CD4, CD25 and FoxP3 and the percentage of CD4^+^ CD25^+^ T cells producing cytokines IL-10 and TGF-β was determined by flow cytometry.

### Flow cytometric immunophenotyping

DCs treated as indicated were washed with PBS supplemented with 2 % FCS and 0.1 % sodium-azide, and incubated (1x10^5^ cells) with panel of immunophenotyping antibodies for surface labeling: anti-CD83-FITC, anti-CD86-FITC, anti-CD40-APC, anti-CCR7-FITC, (BioLegend Inc., San Diego, CA, USA), anti-CD14-FITC, anti-HLA-DR-APC Cy7 (Miltenyi Biotec, Bergisch Gladbach, Germany), and anti-Ig-like transcript (ILT)3-PE (R&D Systems, USA), for 30 minutes at 4 °C. For intracellular staining cells were fixed using the flow cytometry fixation and permeabilization kit I (R&D Systems) and intracellular staining was performed with the following antibodies: anti-TGF-β-APC, anti-IL-12p40/p70-PE and anti-IL-10-PE (BioRad). Isotype-matched control monoclonal antibodies were used to determine non-specific background staining immunoglobulin (Ig)G1a negative control-peridinin-chlorophyll-protein complex (PerCP), IgG1 negative control-phycoerythrin (PE), IgG1 negative control-fluorescein isothiocyanate (FITC), IgG1a negative control-PECy5, IgG1 negative control-allophycocyanin (APC). Labeled cells were analyzed on BD FACS LSR II flow cytometer and data were analyzed using FlowJo software.

T cells were stained for surface markers with the following antibodies: anti-CD4-FITC, anti-CD4-APC (eBioscience, San Diego, CA, USA), anti-CD25-PeCy5 (BD Pharmigen, CA, USA) antibodies. After cell fixation and permeabilization, the following mAbs for intracellular staining were used: anti forkhead box Foxp3-PE, anti-TGF-β-APC, anti-IL-4-PE, anti- IL-10-PE (eBioscience), anti-IFNγ-FITC (R&D Systems). The gates for cultivated DC and T cells were set according to their specific forward scatter (FS) and side scatter (SS) properties, thereby avoiding dead cells with low FS/SS signal. A minimum of 5000 cells was acquired per sample at a flow rate ranging between 50-100 cells/s. Data were acquired using a BD FACS LSR II flow cytometer and analyzed using FlowJo software.

### Proteomic analyses

The label-free in solution proteomic analysis was performed by liquid chromatography tandem mass spectrometry (LC-MS/MS) by the Proteomics and Mass Spectrometry Core Facility of the University of Bern, Switzerland. Samples were digested (Braga-Lagache et al., 2016[3]) and analyzed by LC-MS/MS as described before (Heller et al., 2020[19]). The identified peptides were blasted against a database for *T. spiralis* (https://parasite.wormbase.org/Trichinella_spiralis_prjna12603) and the UniProt database. From the list of identified peptides, contaminating human proteins were removed. Acceptance criteria for confirmation of protein identity were at least two identified peptides per protein. Functional characterization of isolated protein sequences was based on Gene Ontology (GO) Annotation. For proteins with no annotated function, functional domains were predicted by online analysis using NCBI database. 

Multiple sequence alignment of sequences identified by mass spectrometry and galectins from other organisms was carried out by on-line Clustal X multiple sequence alignment program (Larkin et al., 2007[33]). Galectin sequences from *T. spiralis* (EFV62290), *T. nativa* (KRZ49476), *T. murrelli* (KRX35770.1), *T. nelsoni* (KRX15368.1), *T. patagoniensis* (KRY16872.1), *T. pseudospiralis* (KRY89534.1), *Trichinella sp. T6* (KRX72992.1), *Trichinella sp. T8* (KRZ90044.1), *Trichinella sp. T9* (KRX52357.1), *T. zimbabwensis* gal lec-3 (KRZ13804.1), and the other nematodes *Trichostrongylus colubriformis* (AAD11971.1), *Caenorhabditis elegans* (AAB87718.1), *Caenorhabditis elegans* (NP_496159.1), *Brugia malayi* (AAF37721.1), *Haemonchus contortus* (AAB88823.1), *Dirofilaria immitis* (AAF37720.1), *Wuchereria bancrofti*, partial (EJW88921.1), as well as human gal-1 (P09382) and mouse gal-1 (P16045) were downloaded from NCBI. Obtained protein sequences were used as queries in search for carbohydrate recognition domain (CRD) homology with the above-listed galectin sequences, using BLAST analysis.

### Statistical analysis

One-way analysis of variance (ANOVA) was performed followed by the Bonferroni's multiple comparison test or Tukey's posttest, to analyze differences in means between different groups of treated cells and control groups (GraphPad Prism version 5.00 for Windows, GraphPad Software, San Diego, CA, USA). Data are presented as means ± SD, and differences were considered significant at p values of ≤ 0.05.

## Results

### Identification of proteins reactive with anti-human galectin-1 antibody among T. spiralis muscle larvae antigens

**Figure 1:**
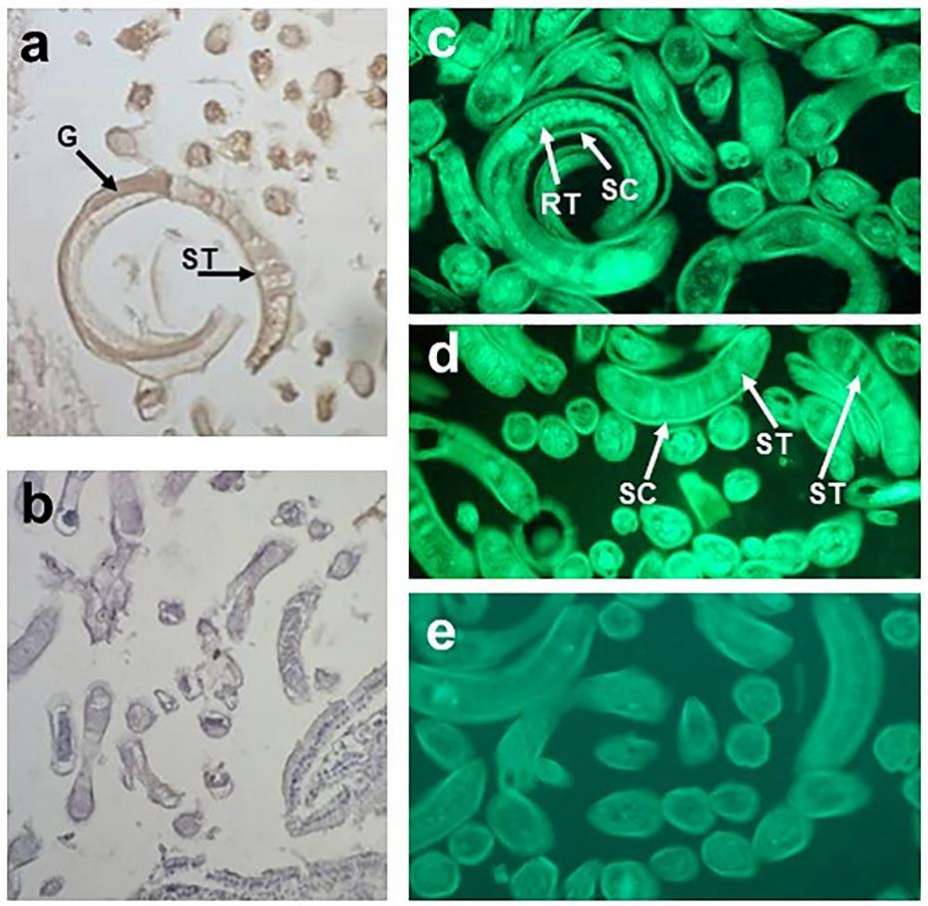
Anti-galectin-1 Ab-reactive proteins in *T. spiralis* muscle larvae. Immunohistochemical (a) and indirect immunofluorescence (IIF) staining (c, d) of the sections of isolated *T. spiralis* muscle larvae with anti-human galectin-1 Ab and (b, e) negative control, without anti-galectin-1 Ab. Arrows indicate binding of anti-galectin-1 Ab to subcuticular region - SC, stichocytes - ST, reproductive tracts - RT and gut - G

**Figure 2:**
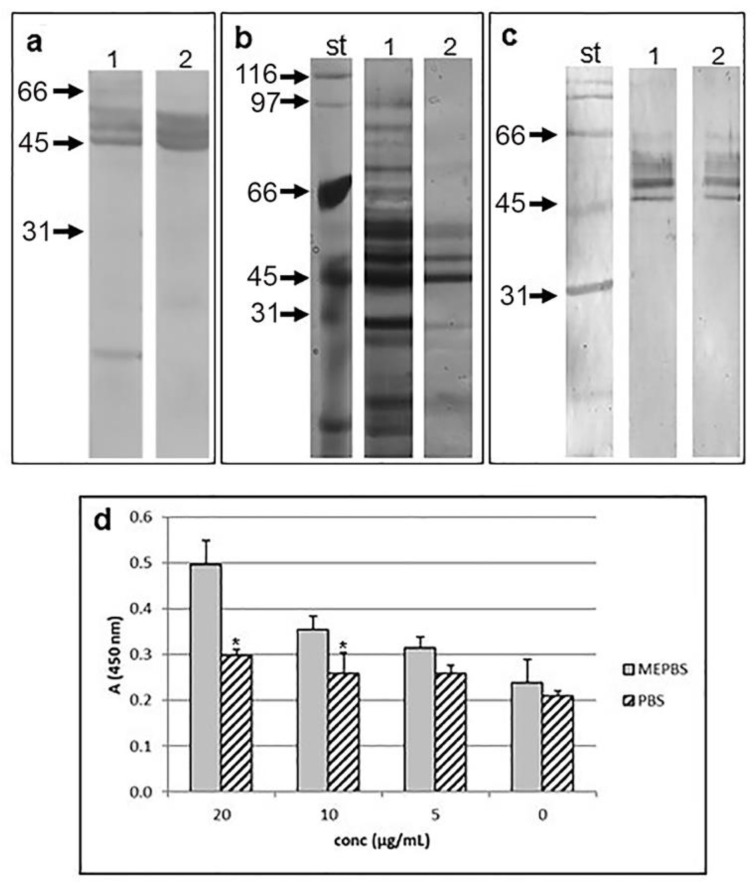
*T. spiralis* proteins recognized by anti-galectin-1 antibodies. Western blot of *T. spiralis* crude extract (a, line 1) and ES L1 products (a, line 2) with anti-human galectin-1 Ab. Mw markers are indicated by arrows. (b) SDS-PAGE of the components of ES L1 isolated on Lactose-Sepharose 4B column. st - Mw standards; line 1: ES L1 proteins; line 2: Lactose eluted proteins. Proteins were stained with silver staining kit. (c) Western blot of lactose eluted proteins with anti-human galectin-1 Ab-HRP (line 1) and anti-mouse galectin-1 Ab-HRP (line 2); st - Mw standards. (d) Binding of TsGal-1-like proteins to Matrigel. Serial dilutions of TsGal-1-like proteins in MePBS or PBS (0-20 μg/ml) were added to microtiter plate wells coated with Matrigel. Bound TsGal-1-like proteins were detected with anti-galectin-1 Ab, followed by anti-rabbit IgG-HRP.

Immunohistochemical and immunofluorescence analysis of *T. spiralis* muscle larvae revealed structures in the subcuticular region, reproductive tract, lower part of the gut and stichosome (organ where ES L1 products are produced) that were recognized by anti-galectin-1 antibody (Figure 1a, c, d(Fig. 1)). Figure 1b and e(Fig. 1) represent non-specific binding, obtained by omitting primary antibodies (negative control). Western-blot analyses of *T. spiralis* crude extract, representing soluble fraction of the whole muscle larvae (Figure 2a, line 1(Fig. 2)), and ES L1 antigens (Figure 2a, line 2(Fig. 2)), performed to determine which parasitic component reacts with anti-galectin-1 antibodies, revealed existence of a certain number of reactive proteins predominantly ranging between 40 and 70 kDa (Figure 2a(Fig. 2)) that could have sequence and/or structural similarities with galectin-1.

### Isolation of TsGal-1-like molecules from T. spiralis ES L1 products

Affinity chromatography of ES L1 antigens on Lactose-Sepahrose 4B, under reducing conditions (which potentiate the lectin activity of galectin family) resulted in the isolation of specific fractions of proteins. Components adsorbed on the column were specifically eluted with lactose, which indicated their lectin activity and carbohydrate binding specificity. Since they were bound to immobilized lactose in the presence of EDTA in the binding/eluting buffer, these proteins do not require metal ions for their saccharide binding. Isolated molecules were separated by SDS-PAGE and visualized by silver staining (Figure 2b(Fig. 2)). Proteins that were isolated from ES L1 products based on their carbohydrate specificity towards lactose reacted with antibodies raised against human and mouse galectin-1 at 49, 58, 62 and 66 kDa, which confirmed their possible sequence and/or structural similarity with galectin-1 (Figure 2c(Fig. 2)).

### Binding assay

Lectin binding activity of TsGal-1-like proteins was tested in a binding assay with Matrigel coated plates. Serial dilutions of TsGal-1-like proteins in MePBS were added to the plates and resulted in dose-dependent binding (Figure 2d(Fig. 2)). When TsGal-1-like proteins were diluted in PBS, the level of binding was significantly lower than binding under reducing conditions, which could be the consequence of inactive CRD in oxidized form. Inhibition of binding with 0.2 M lactose was not accomplished.

### TsGal-1-like proteins induce a semi-mature status of human DCs

**Figure 3:**
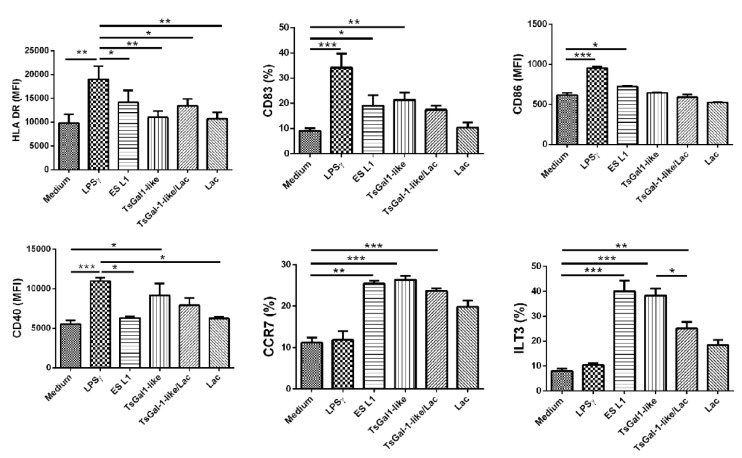
Phenotype of DCs treated with TsGal-1-like proteins. Immature DCs were treated with *T. spiralis* ES L1 antigens (50 μg/ml), TsGal-1-like proteins (5 μg/ml) and LPS/IFNγ and the expression of maturation markers was analyzed by flow cytometry. The summarized results of HLA-DR, CD83, CD86, CD40, CCR7 and ILT3 expression by DCs are shown as mean ± SD from three different experiments. (Representative experiment for the expression of maturation markers is given in Supplementary Figure 1). *p < 0.05, **p < 0.01, ***p < 0.005 compared as indicated (one-way ANOVA with Tukey's posttest).

Our previous studies revealed the potential of *T. spiralis* ES L1 to induce a semi-mature phenotype and tolerize human DCs (Ilic et al., 2018[27]). Since human galectin-1 is suggested to play a role in enhancing DCs tolerogenic potential (Mascanfroni et al., 2011[39]), we tested the potential of TsGal-1-like proteins for the induction of tolerogenic DCs. The effects of these proteins, isolated from ES L1 were compared to the impact of the whole ES L1, while LPS/IFN-γ served as full-maturation stimulus. Immature DCs (CD1a positive, Supplementary Figure 1) were incubated with TsGal-1-like proteins, ES L1 and LPS/ IFN-γ, and examined for the expression of surface markers (representative plots are shown in Supplementary Figure 2). The expression of HLA-DR and CD40 was not affected with ES L1 treatment, while the expression of CD83, CD86, CCR7 and ILT3 was significantly higher compared to control cells, cultivated in medium only (Figure 3(Fig. 3)). TsGal-1-like proteins did not influence the expression of HLA-DR, CD86 and CD40, however, they induced significantly elevated expression of CD83, CCR7 and ILT3. Since it has been suggested that galectin-1 binds to cell surface glycoproteins via CRD, we attempted to demonstrate that TsGal-1-like proteins affect dendritic cell phenotype through a lectin-type interaction that may be inhibited by specific sugar lactose. Only the increased expression of ILT3, a marker of tolerogenic DCs, was diminished by 0.1 M lactose. Thus, the observed phenotype showed that TsGal-1-like proteins induced semi-mature DCs, at least in part through carbohydrate-specific interactions. In contrast to ES L1 and TsGal-1-like-treated DCs, LPS treated DCs exerted upregulated expression of all surface markers, except for CCR7 and ILT3, as expected for type 1 inflammatory stimulus.

**Figure 4:**
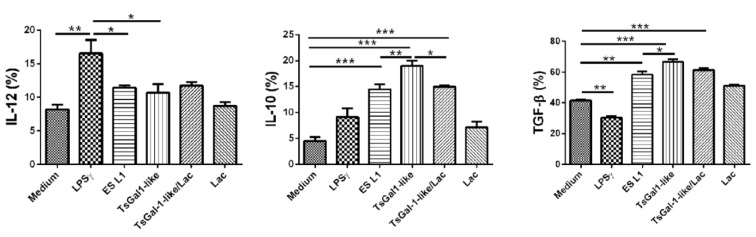
Cytokine expression of TsGal-1-like proteins-pulsed DCs. The summarized results of IL-12p40/p70, IL-10, and transforming growth factor (TGF)-β expression are presented as mean value of percentage (%) of the expression ± SD from three different experiments. *p < 0.05, **p < 0.01, ***p < 0.005 compared as indicated (one-way ANOVA with Tukey's posttest).

Further following the impact of TsGal-1-like proteins on DCs, we analyzed the cytokine production by determining the percentage of IL-10, IL-12, and TGF-β positive DCs by intracellular staining (Figure 4(Fig. 4)). The levels of IL-12 in TsGal-1-like protein and ES L1-treated DCs were unchanged compared to control immature DCs, which means that both stimuli had no influence on the production of IL-12 by DCs. However, both *T. spiralis* products significantly increased the percentage of IL-10^+^ and TGF-β^+^ DCs, compared to control ones. Lactose significantly inhibited the impact of TsGal-1-like proteins only on IL-10 production.

### DCs stimulated with TsGal-1-like proteins induce Th2 and regulatory responses

**Figure 5:**
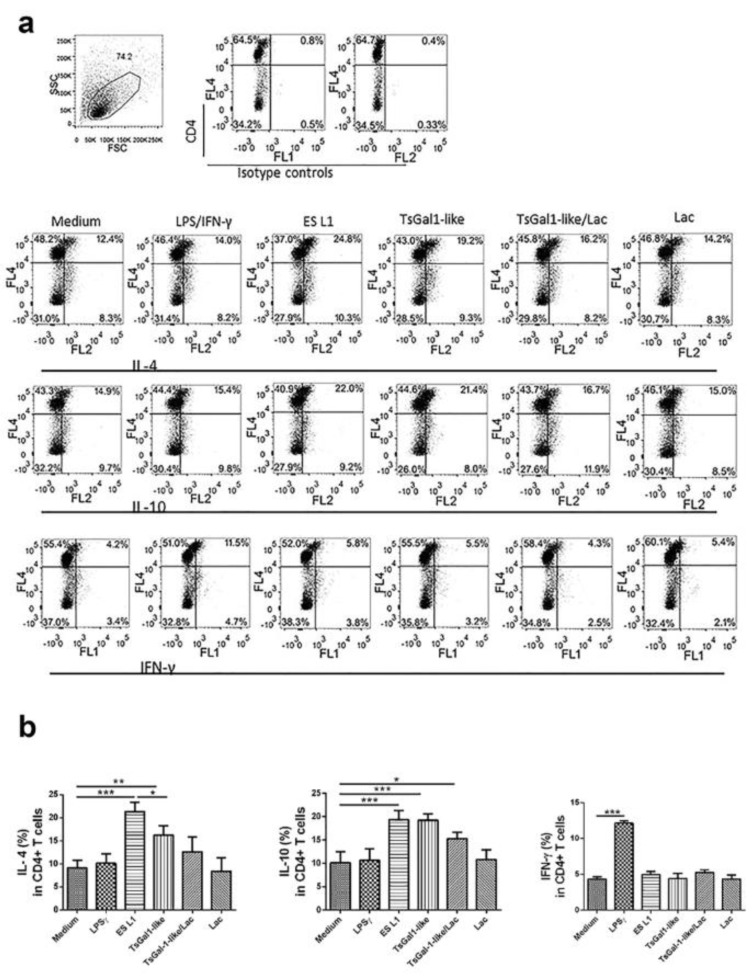
T helper polarization influenced by treated DCs. Treated DCs were thoroughly washed and then cocultured with magnetic-activated cell sorting-purified allogenic T cells (1 × 10^5^/well) for 6 days in 1:20 DC:T cell ratio. (a) Representative analysis of IL-4^+^, IL-10^+^ and IFN-γ^+^ cells within CD4^+^ T cells from one experiment are shown. (b) Presented are the summarized results shown as mean % ± SD of three experiments with different DCs donors. *p < 0.05, **p < 0.01, ***p < 0.001 compared to control, or as indicated by line (one-way ANOVA with Tukey's posttest).

T cell polarizing capacity of DCs, matured under the influence of ES L1 or TsGal-1-like proteins as its components, was evaluated by analyzing the expression of IL-4, IL-10 and IFN-γ in CD4^+^ T cells, after DC/T cell cocultivation. The results indicated that both *T. spiralis* products induce Th2 and regulatory responses, characterized by significantly elevated percentage of IL-4^+^ and IL-10^+^ T cells, compared to controls (Figure 5(Fig. 5)). These DCs however displayed impaired capacity to induce the production of Th1 cytokine IFN-γ, the level of which was at the level of control. The influence of TsGal-1-like proteins was not abolished by preincubation with lactose, just diminished, which indicates that impact of TsGal-1-like proteins on DCs and consequently on T cell polarization could be accomplished with both lectin activity and protein-protein interactions.

**Figure 6:**
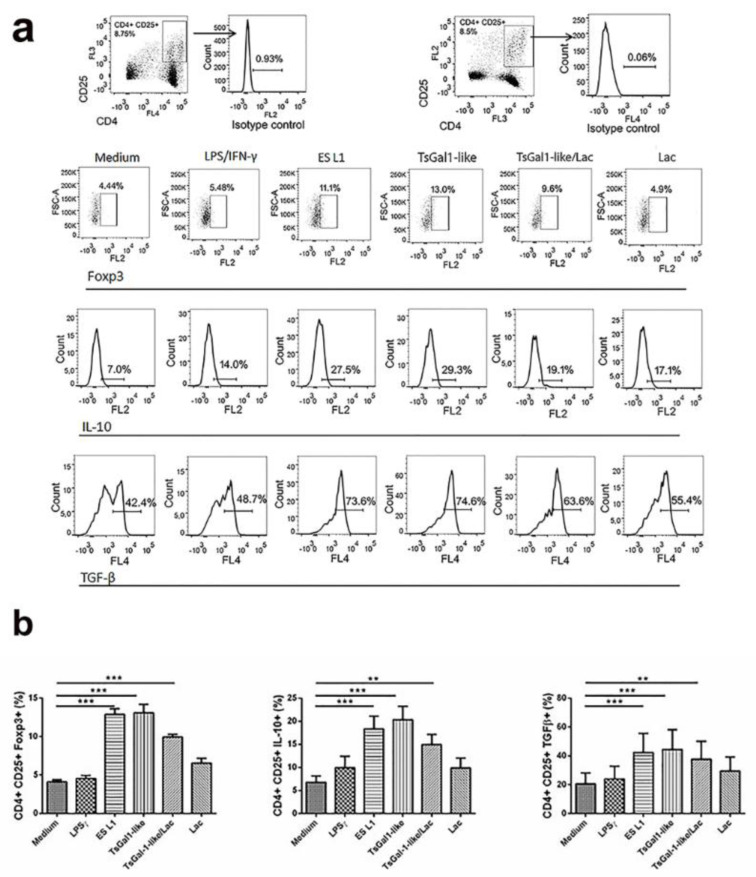
Tolerogenic properties and functions of TsGal-1-like proteins-treated DCs. DCs treated with ES L1 antigens (50 μg/ml), TsGal-1-like proteins (5 μg/ml) and LPS/IFNγ were washed thoroughly and then cocultured with magnetic-activated cell sorting-purified allogenic T cells (Tly) (1 × 10^5^/well) for 3 days in 1:50 DC:T cell ratio and then re-stimulated with interleukin (IL)-2 (2 ng/ml) for another 3 days. (a) Representative analysis of CD25^+^Foxp3^+^ cells within CD4^+^ T cells and IL-10 and transforming growth factor (TGF)-β within CD4^+^CD25^+^ T cell population is shown, (b) the summarized results for the expression of CD4^+^CD25^+^FoxP3^+^ T regulatory cells and IL-10 and TGF-β within CD4^+^CD25^+^ T cells are shown as the mean % ± SD from three different experiments. *p < 0.05, **p < 0.01, ***p < 0.005 compared as indicated by line (one-way ANOVA with Tukey's posttest).

Tolerogenic properties of DCs could be clearly revealed by assessing the presence of Tregs in DCs/T cell coculture. The percentage of Tregs was determined based on their expression of CD25 and FoxP3 within CD4^+^ T cells. TsGal-1-like proteins and ES L1-treated DCs induced an increase in the percentage of CD4^+^CD25^+^Foxp3^+^ compared to control cells (Figure 6a, b(Fig. 6)). These CD4^+^CD25^+^ T cells showed significantly increased expression of IL-10 and TGF-β, anti-inflammatory and regulatory cytokines, compared to T cells primed with control DCs (Figure 6a, b(Fig. 6)). Preincubation with lactose, performed to inhibit interaction of TsGal-1-like proteins with DCs resulted in decreased expression of ILT3 and the production of IL-10, which led to a (non-significant) reduction of the production of IL-10 and TGF-β within CD4^+^CD25^+^ T cells.

### Identification of proteins of Trichinella spiralis using mass spectrometry

The fraction isolated from ES L1 products on Lactose-Sepharose column was analyzed by using mass spectrometry. Obtained protein sequences were used as queries in BLAST search against the WormBase ParaSite and UniProt database (Table 1(Tab. 1)). Seven proteins with annotated function were identified: “Multi cystatin-like domain protein”; “Conserved cysteine-glycine protein 2”; “43 kD secreted glycoprotein” with deoxyribonuclease II domain; “Transmembrane protease serine 9” with two trypsin-like serine protease domains, belonging to a Protease S1 family; “Antigen targeted by protective antibodies”, Secreted from muscle stage larvae 3; and “Conserved cysteine-glycine protein 2-like protein”. Other identified proteins were not annotated and are therefore designated as uncharacterized proteins, although for most of them functional domains could be predicted based on BLAST analysis of NCBI database (Table 1(Tab. 1)). Uncharacterized proteins with accession numbers EFV59180, EFV57884, EFV56954 and EFV57881 possess domains with assigned trypsin-like serine protease activity and were designated as putative trypsin. Protein with accession number EFV51118 possesses CAP (cysteine rich) domain and in NCBI database it is recognized as putative venom allergen 5, while according to UniProt database it has 99.1 % identity with Glioma pathogenesis-related protein 1. According to NCBI database EFV50690 is similar to putative pyroglutamyl peptidase 1, while EFV56088 is similar to retinoblastoma binding protein 6.

The homology comparison of amino acid sequences of proteins identified in TsGal-1-like protein isolate with galectin sequence of *T. spiralis*, several nematodes, mouse and human revealed that none of the identified proteins were actually galectins. However, comparing amino acid sequences of proteins from TsGal-1-like isolate with sequences of CRD domains revealed matches in key residues of the sugar binding sites in five proteins, although none of these proteins matches all amino acids important for binding. The 43 kDa secreted glycoprotein (EFV58105) with calculated Mw 37.7 kDa, possesses similarity with the β-galactoside binding site sequence of human and mouse galectin-1. Amino acid sequence of Transmembrane protease serine 9 (EFV62279), with calculated Mw 71.6 kDa, matches with sugar binding site of *C. elegans* galectin, and human and mouse galectin-1. Amino acid sequence of Antigen targeted by protective antibodies (EFV51108), calculated Mw 36.3 kDa, has similarity with *C. elegans* and *T. spiralis* galectin. Uncharacterized protein EFV59180, designated in NCBI database as putative trypsin, possesses similarity within sugar binding domain of *T. spiralis* galectin. Uncharacterized protein EFV57881, calculated Mw 53.4 kDa, designated in NCBI database also as putative trypsin possesses similarity within sugar binding domain of *T. spiralis*, *T. circumcincta*, *C. elegans*, *W. bancrofti* galectins, as well as with human and mouse galectin-1.

See also the Supplementary data.

## Discussion

Like other parasites *T. spiralis* developed mechanisms to survive in the host organism, which includes induction of regulatory immune responses responsible for maintenance of homeostasis (Ilic et al., 2012[28]). Key modulators of the host immune system are present in *T. spiralis* ES L1 products that make an interface between the muscle larvae and the host, but the exact components are still elusive. The investigations in this study started from the assumption that some of these immunomodulatory molecules responsible for the induction of tolerogenic environment might be members of a galectin family, galectin-1-like proteins, since it was shown that galectin-1 is implicated in the regulation of innate and adaptive immune responses, playing essential roles in the resolution of inflammation (Sundblud et al., 2017[53]). It was shown that galectin-1-defficient mice had dysfunctional Tregs, exhibited increased production of pro-inflammatory cytokines and are susceptible to development of autoimmune diseases. Recombinant galectin-1 proved to be efficient in ameliorating inflammatory diseases such as type-1 diabetes (Perone et al., 2009[42]) and rheumatoid arthritis (Rabinovitch et al., 1999[44]). Since galectins share conserved sequences responsible for carbohydrate binding (Hirabayashi and Kasai, 1994[22]), we assumed that due to the sequence or structural homology, *T. spiralis* proteins could be recognized by antibodies raised against human galectin-1. Indeed, structures in the subcuticular region, reproductive tract and stichosome of *T. spiralis* muscle larvae, as well as some of its components from the crude extract and ES L1 products reacted with anti-human galectin-1 antibodies. The existence of proteins with lectin activity was confirmed by affinity chromatography isolation and by Matrigel binding assay. Matrigel consists of molecules present in extracellular matrix (as laminin and fibronectin) that are natural ligands for galectin-1 (Camby et al., 2006[4]). Specific binding of galectin-1 to glycan ligands depends on the oxidative state of cysteine residues necessary for dimerization, i.e., oxidation inactivates carbohydrate binding function (Stowell et al., 2009[52]). We have found that binding of TsGal-1-like proteins accomplished in the reducing conditions that prevented oxidative inactivation, was diminished in PBS, but the presence of lactose did not inhibit the initial binding. The reason could be low affinity binding of galectin-1 CRD to individual lactosamine units, especially in the solution (Ahmad et al., 2004[1]). Affinity for binding increases when lactosamine disaccharides are arranged in multimer chains or when they are attached to surfaces, like in extracellular matrix (He and Baum, 2004[18]).

During chronic *T. spiralis* infection, the impact on the host immune system is mediated through the interaction of parasite ES L1 product and host immune cells, DCs in particular. Our previous findings revealed the capacity of *T. spiralis* ES L1 products to induce stabile/stable tolerogenic DCs, capable of polarizing T-cell response towards Th2 and regulatory type (Ilic et al., 2018[27]). Dendritic cells treated with TsGal-1-like proteins acquire tolerogenic phenotype characterized by low expression of HLA-DR, CD86 and CD40, and high expression of CD83, CCR7 and ILT-3, together with increased production of anti-inflammatory cytokines IL-10 and TGF-β and low production of inflammatory cytokine IL-12. TsGal-1-like proteins completely repeated the effect of the whole ES L1. The expression of ILT-3 clearly points to the tolerogenic properties of TsGal-1-like stimulated DCs (Vlad et al., 2010[57]), as does the increased ratio of IL-10/IL-12 in DCs (Raker et al., 2015[47]). High expression of CCR7, along with increased production of IL-10, is required for the migration of DCs to lymph nodes and induction of Tregs (Forster et al., 2008[12]). Tolerogenic capacity of TsGal-1-like proteins stimulated DCs was confirmed by their ability to induce expansion of CD4^+^CD25^+^Foxp3^+^ Tregs in DC/T cell co-culture. Synergistic effects of IL-10 and TGF-β were implicated in the development of DC with tolerogenic properties, capable of inducing IL-10-producing Tregs (Torres-Aguilar et al., 2010[54]), which we found to be increased in the percentage in a co-culture with TsGal-1 like proteins treated DCs. There is no doubt that TsGal-1-like proteins can mimic some of the immunomodulatory functional properties of human galectin-1 (Ilarregui et al., 2009[25]). 

However, proteomic analysis of the TsGal-1-like isolate from ES L1 did not detect galectins. Among nineteen proteins identified by MS analysis, none shared homology with human galectin-1. Lactose bound fraction comprised a mixture of different proteins, predominantly proteases. Protein sequence similarity search revealed presence of several proteins with trypsin-like serine protease domain, marked as putative trypsin, with different calculated molecular masses, proteins with serine protease domain, deoxyribonuclease II catalytic domain, multi cystatin-like domain protein. 

ES L1 products are involved in the invasion and developmental process of *T. spiralis* (reviewed in Sofronic-Milosavljevic et al., 2015[50]), and also play an important role in immunomodulation through interaction with immune cells (Ilic et al., 2018[27]; Gruden-Movsesijan et al., 2011[17]). Cystatins in parasitic nematodes, besides their role in inhibition of cysteine proteases activity, are involved in the modulation of the host immune response (Klotz et al., 2011[31]). Multi cystatin-like domain protein, identified in a lactose-specific isolate is ubiquitously distributed throughout *Trichinella spp*., which indicates its importance for their life cycle. This protein was first identified by Robinson et al. (2007[48]), as a non-inhibitory cystatin MCD-1, present in the muscle larvae, with a probable role in triggering host immune response. Another cystatin was detected searching the *T. spiralis* genome-cystatin-like protein (Ts-cystatin) that plays an important role in *Trichinella* resistance to rapid expulsion, but also in the invasion process (Liu et al., 2014[37]). The authors also found that Ts-cystatin induced a Th1/Th2-mixed type of immune response, by increasing expression of T-bet and GATA3. T-bet and GATA3 transcription factors are implicated in Th1 and Th2 differentiation, respectively. Kobpornchai and colleagues (2020[32]) recently identified and characterized a novel *T. spiralis* cystatin (TsCstN), which inhibits inflammation mediated by LPS-treated macrophages. Sequence homology search revealed that our uncharacterized protein EFV50947, with calculated molecular weight of 13.2 kDa, is in fact this novel cystatin.

Another protein with annotated function in TsGal-1 like isolate is conserved cysteine-glycine protein 2, a member of a family of proteins with conserved cysteine-glycine rich domain (CCG), specific for nematodes. Two proteins belonging to this family were identified in *T. spiralis*, coded by two genes, constitutively expressed *Ts-ccg-1* and *Ts-ccg-2*, expressed only in the muscle larvae (Gare et al., 2004[14]). Ts-CCG-2 protein, found in excretory-secretory products of the muscle larvae is identical to the protein isolated in this study. The function of CCG proteins is still unknown, but since Ts-CCG-2 protein is developmentally regulated and expressed only in the muscle larvae, it was proposed that it is probably involved in the nurse cell formation (Gare et al., 2004[14]). 

Among proteins of muscle larvae ES L1 products that were recognized by *Trichinella* positive sera (Sofronic-Milosavljevic et al., 2005[51]; Gómes-Morales et al., 2012[15]), a protein with deoxyribonuclease II activity was identified (Wang et al., 2014[58]). This protein was first discovered by Vassilatis et al. (1992[56]) as P43, and this is the same protein we identified in the isolate as 43 kDa secreted glycoprotein. The role of secreted DNAse II from *T. spiralis* muscle larvae could be the cleavage of undigested DNA that remains in the circulation due to inadequate removal of the apoptotic cells emerged as a consequence of *T. spiralis* invasion. Exogenous DNA can activate innate immunity and trigger mechanisms that may end in the development of autoimmune diseases, while the action of DNAse II could reduce the chances for the development of inflammatory responses and on that way preserve homeostasis (Liu et al., 2008[35]).

Serine proteases are a superfamily of proteolytic enzymes that are present in various isoforms in all developmental stages of *T. spiralis*, which indicates their importance for larval growth and development (Yang et al., 2015[64]). Serine proteases are represented by several members identified in ES L1 proteins of *T. spiralis* muscle larvae (Wang et al., 2014[58]). These proteins are mostly glycosylated, some of them bearing a highly antigenic sugar moiety, tyvelose, which elicits protective antibodies in the host organism, pointing to the importance of tyvelose-bearing glycoproteins for establishing parasitism (Romaris et al., 2002[49]). Serine proteases secreted by *T. spiralis *muscle larvae are involved in larval invasion, molting, digestion and proteolysis (Yang et al., 2015[64]). Our investigation identified six proteins with predicted serine protease trypsin domain: transmembrane serine protease 9, antigen targeted by protective antibodies and four uncharacterized proteins (EFV59180, EFV57884, EFV56954 and EFV57881). Transmembrane serine protease 9, discovered by Trap and colleagues (2006[55]), possesses two serine protease trypsin-like domains within a single protein, which is the unusual feature described by these authors for the first time in parasites. Based on the constitutive expression and localization of this serine protease, the authors suggested a function in molting and/or digestion in *Trichinella spp*. BLAST sequence analysis revealed that antigen targeted by protective antibodies with trypsin-like serine protease domain (EFV51108) is 100 % identical to 31 kDa serine protease (Ts31) characterized by Cui et al. (2015[6]), which was also named trypsin-like 45 kDa antigen (Wang et al., 2013[59]). Uncharacterized protein EFV57881 was found to be identical to the putative trypsin (XP_003376874.1) presented by Wang et al. (2014[58]), and 86.7 % identical to putative serine protease TspSP-1, described by Romaris et al. (2002[49]). Putative trypsin EFV57884 (according to the NCBI database) is identical to the putative trypsin (XP_003376871.1) found by Wang et al. (2014[58]), while uncharacterized protein EFV56954 possesses 82.7 % identity with muscle larvae putative serine protease TsSP (GenBank accession no. ABY60762) (Liu et al., 2015[36]). The impact of *T. spiralis* serine proteases on the immune response of the host is scarcely investigated. It was shown that vaccination with recombinant TsSP induced a Th2 immune response and production of anti-rTsSP antibodies that confer partial protection against challenge infection (Liu et al., 2015[36]), while recombinant serine protease from *T. spiralis* adults provoked a mixed Th1/Th2 response with elevated production of IFN-γ, IL-4 and IL-10 (Xu et al., 2021[62]). Recombinant serine protease from adult stage of *T. spiralis* was able to suppress the Th1 and Th17 immune response and potentiate the Th2 and Treg response, exerting beneficial effect on TNBS-induced mouse colitis (Pang et al., 2020[41]). Proteins with serine protease trypsin domain are dominant in the TsGal-1-like fraction, so we can assume that these molecules could have the strongest impact on T-cell polarization in DC/T-cell co-cultures. Increased production of IL-4 and IL-10 in T cells cultivated with TsGal-1-like stimulated DCs was indeed observed, while there was no effect on the production of IFN-γ. The role of individual proteins from TsGal-1-like isolate is yet to be discovered, however, it was documented that some of them possess immunodominant epitopes and can elicit immune response.

The intriguing finding of this study was that none of the isolated proteins possessed homology with known galectins, and yet some of them have been recognized by anti-human and anti-mouse galectin-1 antibodies. Sequence alignment of identified proteins with galectins from *Trichinella spp*. and other nematodes, and with mouse and human galectin-1, revealed that some of these proteins with lactose-binding property share amino acids with sugar binding sequence motif characteristic for galectins. It is possible that the existence of sequences with high percentage of identity with CRD domain of human and mouse galectin-1 that were detected within 43 kDa secreted protein, transmembrane protease serine 9 and uncharacterized protein designated as putative trypsin, allows for a structural fold that resembles a galectin-1 topology and on that way provides a binding site for anti-mouse and anti-human galectin-1 antibodies. The fact that the identified proteins were isolated based on specific binding to lactose raises questions about structures that are involved in sugar binding and what is the significance of this unusual feature that we observed. 

## Notes

Natasa Ilic and Zanka Bojic-Trbojevic contributed equally as first author.

## Declaration

### Conflict of interest

The authors declare that they have no conflict of interest.

### Acknowledgments

We thank the Proteomics and Mass Spectrometry Core Facility of the University of Bern, Switzerland, for analysis of protein samples. This work was supported by the Ministry of Education, Science and Technological Development, Republic of Serbia (Project no. 173047 and 173004; contract no. 451-03-68/2022-14/200019).

## Supplementary Material

Supplementary information

Supplementary data

## Figures and Tables

**Table 1:**
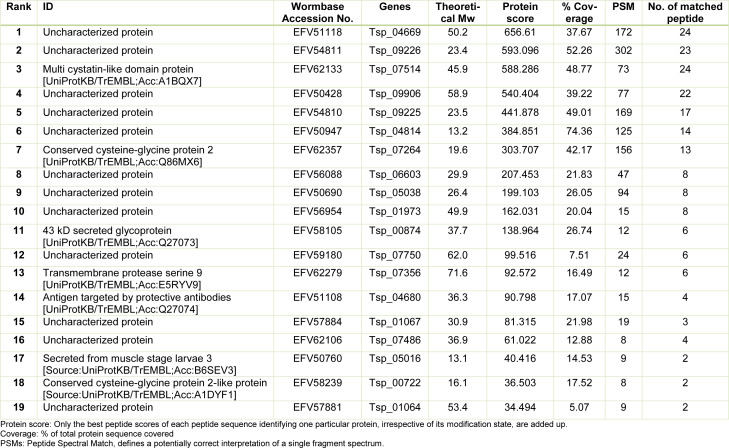
Identification of proteins in ES L1 fraction by mass spectrometry
