# Supplementary material for: Immunomodulatory components of Trichinella spiralis excretory-secretory products with lactose-binding specificity
Source: EXCLI J. 2022 Jun 3;21:793–813. doi: 10.17179/excli2022-4954 (PMC9360477; doi:10.17179/excli2022-4954)
Supplement: Supplementary information [file EXCLI-21-793-s-001.pdf]

EXCLI Journal 2022;21:793-813 – ISSN 1611-2156
Received: April 14, 2022, accepted: May 31, 2022, published: June 03, 2022

Supplementary information to:

Original article:

IMMUNOMODULATORY COMPONENTS OF *TRICHINELLA SPIRALIS* EXCRETORY-SECRETORY PRODUCTS WITH LACTOSE-BINDING SPECIFICITY

Natasa Ilic[1#], Zanka Bojic-Trbojevic[1#], Britta Lundström-Stadelmann[2], Danica Cujic[1], Ivana Mitic[1], Alisa Gruden-Movsesijan[1*]

[1] University of Belgrade, Institute for the Application of Nuclear Energy, Banatska 31b, 11080 Belgrade, Serbia
[2] Institute of Parasitology, Department of Infectious Diseases and Pathobiology, Vetsuisse Faculty, University of Bern, Länggassstrasse 122, 3012 Bern, Switzerland

* **Corresponding author:** Alisa Gruden-Movsesijan, University of Belgrade, Institute for the Application of Nuclear Energy, Banatska 31b, 11080 Belgrade, Serbia. Tel.: +381 641510389; E-mail: alisa@inep.co.rs

\# These authors contributed equally to this work.

https://dx.doi.org/10.17179/excli2022-4954

This is an Open Access article distributed under the terms of the Creative Commons Attribution License (http://creativecommons.org/licenses/by/4.0/).

EXCLI Journal 2022;21:793-813 – ISSN 1611-2156
Received: April 14, 2022, accepted: May 31, 2022, published: June 03, 2022

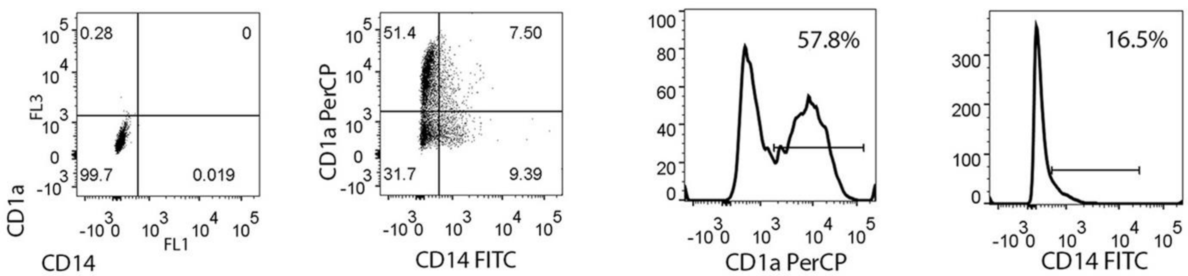

Supplementary Figure 1: Differentiation of dendritic cells (DCs). Immature DCs were generated from monocytes in GM-CSF/interleukin (IL)-4 supplemented medium, during 5 days and the expression of CD14 and CD1a was analyzed by flow cytometry. Differentiation of DCs is shown as a representative plot and histograms from one out of three experiments.

EXCLI Journal 2022;21:793-813 – ISSN 1611-2156
Received: April 14, 2022, accepted: May 31, 2022, published: June 03, 2022

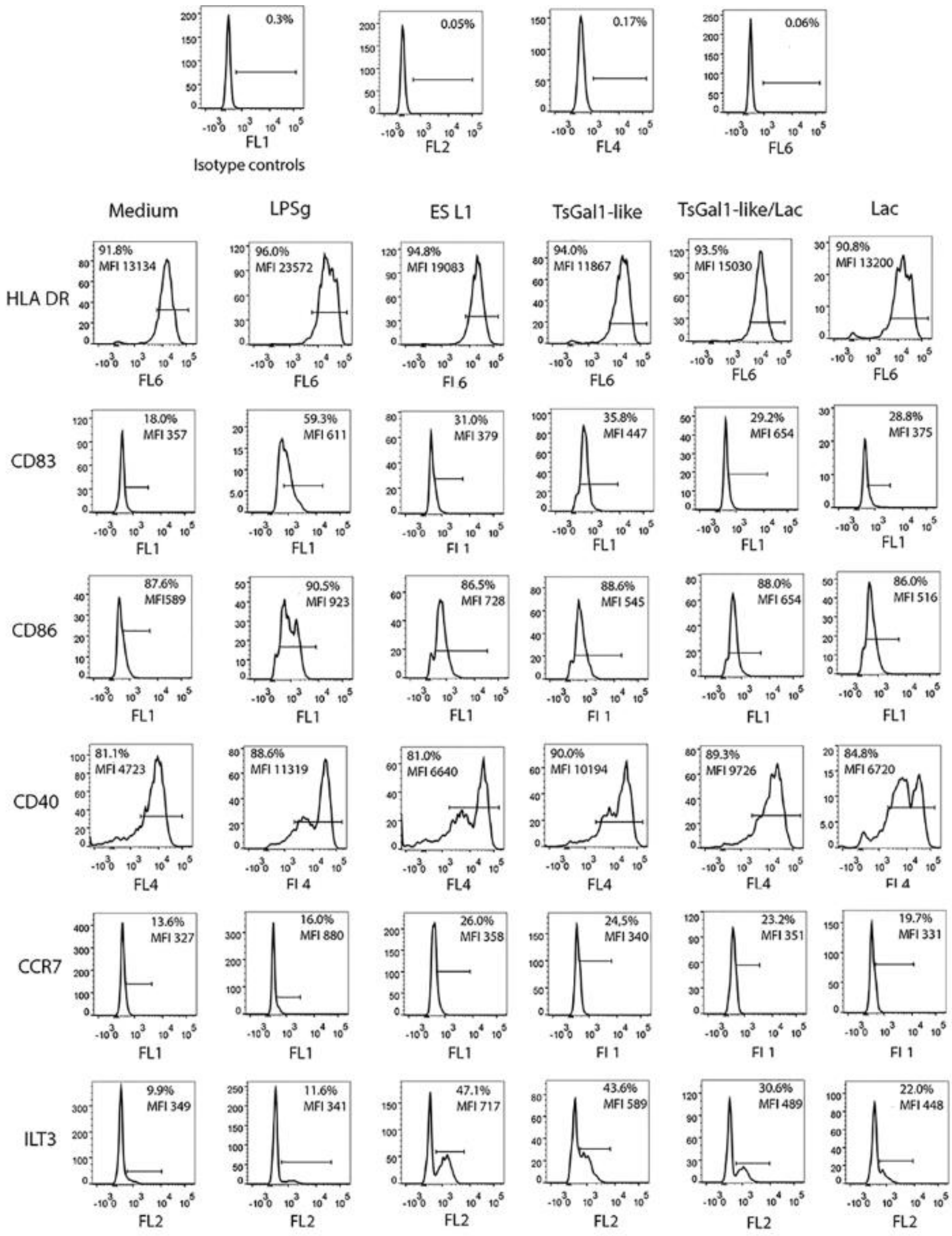

Supplementary Figure 2: Effect of ES L1 and TsGal1-like proteins on the expression of maturation markers on dendritic cells (DCs). Immature DCs were incubated with LPS/IFN-γ, ES L1 (50 µg/ml), TsGal1-like proteins (5 µg/ml), preincubated or not with 0.2 M lactose, and lactose alone. Non-treated cells cultivated in medium were used as a negative control (control) and LPS/IFN-γ (200 ng/ml of LPS and 50 ng/ml IFN-γ) treated DCs were used as a positive control for complete maturation. After 48 h, the expression of markers (HLA-DR, CD83, CD86, CD40, CCR7 and ILT3) was measured by flow cytometry. Representative analysis of surface markers expression, as one out of three performed experiments, is shown.
