# Supplementary material for: Immunomodulatory components of Trichinella spiralis excretory-secretory products with lactose-binding specificity
Source: EXCLI J. 2022 Jun 3;21:793–813. doi: 10.17179/excli2022-4954 (PMC9360477; doi:10.17179/excli2022-4954)
Supplement: Supplementary data [file EXCLI-21-793-s-002.pdf]

EXCLI Journal 2022;21:793-813 – ISSN 1611-2156
Received: April 14, 2022, accepted: May 31, 2022, published: June 03, 2022

Supplementary data to:

Original article:

IMMUNOMODULATORY COMPONENTS OF *TRICHINELLA SPIRALIS* EXCRETORY-SECRETORY PRODUCTS WITH LACTOSE-BINDING SPECIFICITY

Natasa Ilic[1#], Zanka Bojic-Trbojevic[1#], Britta Lundström-Stadelmann[2], Danica Cujic[1], Ivana Mitic[1], Alisa Gruden-Movsesijan[1*]

1 University of Belgrade, Institute for the Application of Nuclear Energy, Banatska 31b, 11080 Belgrade, Serbia
2 Institute of Parasitology, Department of Infectious Diseases and Pathobiology, Vetsuisse Faculty, University of Bern, Länggassstrasse 122, 3012 Bern, Switzerland

* **Corresponding author:** Alisa Gruden-Movsesijan, University of Belgrade, Institute for the Application of Nuclear Energy, Banatska 31b, 11080 Belgrade, Serbia.
Tel.: +381 641510389; E-mail: alisa@inep.co.rs

\# These authors contributed equally to this work.

https://dx.doi.org/10.17179/excli2022-4954

This is an Open Access article distributed under the terms of the Creative Commons Attribution License (http://creativecommons.org/licenses/by/4.0/).

EXCLI Journal 2022;21:793-813 – ISSN 1611-2156
Received: April 14, 2022, accepted: May 31, 2022, published: June 03, 2022

Raw data concerning **Figure 2d**. ***T. spiralis* proteins recognized by anti-galectin-1 antibodies, (d) Binding of TsGal-1-like proteins to Matrigel.** Serial dilutions of TsGal-1-like proteins in MePBS or PBS (0-20 µg/ml) were added to microtiter plate wells coated with Matrigel. Bound TsGal-1-like proteins were detected with anti-human galectin-1 Ab. The summarized results of binding assay are shown as mean absorbance obtained on 450 nm ± SD. *p < 0.05

**Samples (µg/ml)**	**MEPBS (A 450 nm)**	**Mean ± SD**	**PBS (A 450nm)**	**Mean ± SD**
20	0.435<br>0.518<br>0.534	0.496 ± 0.05	0.314<br>0.293<br>0.287	0.298 ± 0.01*
10	0.348<br>0.386<br>0.330	0.355 ± 0.03	0.216<br>0.306<br>0.255	0.259 ± 0.05*
5	0.342<br>0.306<br>0.296	0.315 ± 0.02	0.246<br>0.249<br>0.280	0.258 ± 0.02
0	0.298<br>0.213<br>0.204	0.238 ± 0.05	0.221<br>0.208<br>0.198	0.209 ± 0.01

EXCLI Journal 2022;21:793-813 – ISSN 1611-2156
Received: April 14, 2022, accepted: May 31, 2022, published: June 03, 2022

Raw data concerning **Figure 3**. **Phenotype of dendritic cells (DCs) treated with TsGal-1-like proteins** – the expression of maturation markers, analyzed by flow cytometry, is presented as percentage (%) or Mean fluorescence intensity (MFI) for each of three independent experiments, all upon the treatment of DCs with *T. spiralis* ES L1 components (50 µg/ml), TsGal-1-like proteins (5 µg/ml) and LPS/IFN-γ (200/20 ng/ml) respectively. Non-treated cells were used as controls. The specificity of TsGal-1-like proteins interaction with cell surface glycoproteins was assessed by inhibition of binding with lactose. TsGal-1-like proteins were incubated with an equal volume of 0.2 M lactose (TsGal-1-like/Lac) before introducing in DC culture, and the control for the inhibitory effect of lactose (Lac) on binding were DCs treated with 0.1 M Lac. The summarized results of HLA-DR, CD83, CD86, CD40, CCR7 and ILT3 expression by DCs are shown as mean ± SD from three different experiments. *p < 0.05, **p < 0.01, ***p < 0.005 compared to control untreated DCs (other comparisons are indicated by lines in Figure 3).

Markers on DCs	Treatment	Expression of markers on DCs	Mean ± SD
HLA DR (Expression shown as MFI)	Control	6779<br>13134<br>9556	9823 ± 3185.90
	LPS/IFN-γ	13892<br>23572<br>19540	19001.3 ± 4862.43**
	ES L1	10822<br>19093<br>12669	14194.7 ± 4341.44
	TsGal-1-like	8474<br>11887<br>12670	11010.3 ± 2231.15
	TsGal-1-like/Lac	10305<br>15030<br>14808	13381 ± 2666.21
	Lac	8407<br>13200<br>10409	10672 ± 2407.30
CD83 (Expression shown as %)	Control	7.0<br>10.7<br>9.6	9.1 ± 1.9
	LPS/IFN-γ	23.8<br>42.9<br>35.7	34.1 ± 9.65***
	ES L1	11.9<br>26.5<br>18.8	19.1 ± 7.30*
	TsGal-1-like	17.1<br>27.0<br>20.1	21.4 ± 5.08**
	TsGal-1-like/Lac	14.5<br>20.3<br>17.5	17.4 ± 2.90
	Lac	7.37<br>14.4<br>9.4	10.4 ± 3.62
CD86 (Expression shown as MFI)	Control	589<br>672<br>592	617.7 ± 47.08
	LPS/IFN-γ	923<br>979<br>966	956.0 ± 29.31***
		738	

EXCLI Journal 2022;21:793-813 – ISSN 1611-2156
Received: April 14, 2022, accepted: May 31, 2022, published: June 03, 2022

	ES L1	727<br>707	724.0 ± 15.72*
	TsGal-1-like	645<br>657<br>630	644.0 ± 13.53
	TsGal-1-like/Lac	654<br>550<br>570	591.3 ± 55.18
	Lac	516<br>543<br>520	526.3 ± 14.57
CD40 (Expression shown as MFI)	Control	589<br>562<br>592	617.7 ± 48.07
	LPS/IFN-γ	923<br>979<br>966	956 ± 29.31***
	ES L1	738<br>727<br>707	724 ± 15.72
	TsGal-1-like	645<br>657<br>630	644 ± 13.23*
	TsGal-1-like/Lac	654<br>550<br>570	591.3 ± 55.18
	Lac	516<br>543<br>520	526.3 ± 14.57
CCR7 (Expression shown as %)	Control	9.55<br>13.6<br>10.4	11.2 ± 2.14
	LPS/IFN-γ	8.97<br>16.0<br>10.6	11.9 ± 3.68**
	ES L1	24.1<br>26.5<br>25.5	25.4 ± 1.21***
	TsGal-1-like	24.5<br>27.9<br>26.5	26.3 ± 1.71***
	TsGal-1-like/Lac	23.2<br>23.0<br>24.8	23.7 ± 0.99
	Lac	19.7<br>22.5<br>17.1	19.8 ± 2.70
ILT3 (Expression shown as %)	Control	6.4<br>9.9<br>7.7	8.0 ± 1.77
	LPS/IFN-γ	9.15<br>11.8<br>10.2	10.4 ± 1.33
	ES L1	32.5<br>47.1<br>40.4	40.0 ± 7.31***
		34.7	

EXCLI Journal 2022;21:793-813 – ISSN 1611-2156
Received: April 14, 2022, accepted: May 31, 2022, published: June 03, 2022

	TsGal-1-like	43.6<br>36.7	38.3 ± 4.67***
	TsGal-1-like/Lac	30.2<br>22.0<br>28.9	27.0 ± 4.41**
	Lac	22.1<br>14.8<br>18.2	18.3 ± 3.60

EXCLI Journal 2022;21:793-813 – ISSN 1611-2156
Received: April 14, 2022, accepted: May 31, 2022, published: June 03, 2022

Raw data concerning **Figure 4**. **Cytokine expression of TsGal-1-like proteins-pulsed dendritic cells (DCs)** - the expression of IL-10, IL12p40/p70 and transforming growth factor (TGF)-β analyzed by flow cytometry is presented as percentage (%) for each of three independent experiments, all upon the treatment of DCs with LPS/IFN-γ (200/20 ng/ml), *T. spiralis* ES L1 products (50 µg/ml) and TsGal-1-like proteins (5 µg/ml), with or without 0.1 M lactose (Lac) as inhibitor of binding. Non-treated cells were used as controls. The summarized results are presented as mean value of percentage (%) of the expression ± SD from three different experiments. **p < 0.01. ***p < 0.005 compared to control untreated DCs (other comparisons are indicated by lines in Figure 4).

Cytokines	Treatment	Expression of cytokines in DCs	Mean ± SD
IL-10 (Expression shown as %)	Control	3.36<br>5.85<br>4.4	4.5 ± 1.25
	LPS/IFN-γ	5.91<br>11.4<br>10.1	9.1 ± 2.87
	ES L1	12.84<br>16.2<br>14.4	14.5 ± 1.68***
	TsGal-1-like	17.4<br>20.8<br>18.8	19.0 ± 1.71***
	TsGal-1-like/Lac	15.5<br>15.0<br>14.4	15.0 ± 0.55***
	Lac	5.6<br>92<br>6.6	7.1 ± 1.86
IL-12 p40/p70 (Expression shown as %)	Control	9.4<br>7.8<br>8.1	8.2 ± 1.16
	LPS/IFN-γ	19.5<br>11.5<br>17.4	16.5 ± 3.48**
	ES L1	11.0<br>12.1<br>11.0	11.4 ± 0.61
	TsGal-1-like	9.1<br>13.1<br>9.9	10.7 ± 2.12
	TsGal-1-like/Lac	12.7<br>10.9<br>11.6	11.7 ± 0.91
	Lac	8.1<br>9.78<br>8.3	8.7 ± 0.92
TGF-β (Expression shown as %)	Control	40.6<br>43.0<br>41.1	41.6 ± 1.27
	LPS/IFN-γ	28.7<br>32.0<br>30.3	30.3 ± 1.65**
	ES L1	61.8<br>54.6<br>58.8	58.4 ± 3.62**
		64.2	

EXCLI Journal 2022;21:793-813 – ISSN 1611-2156
Received: April 14, 2022, accepted: May 31, 2022, published: June 03, 2022

	TsGal-1-like	70.0<br>66.2	66.8 ± 2.95***
	TsGal-1-like/Lac	59.4<br>63.6<br>61.1	61.4 ± 2.11***
	Lac	50.6<br>52.4<br>50.8	51.3 ± 0.99

EXCLI Journal 2022;21:793-813 – ISSN 1611-2156
Received: April 14, 2022, accepted: May 31, 2022, published: June 03, 2022

Raw data concerning **Figure 5. T helper polarization influenced by treated DCs** - the expression of IL-4, IL-10 and IFN-γ within the $CD4^+$ T cell population, analyzed by flow cytometry, is presented as percentage (%) for each of three independent experiments, all upon the co-culture of treated DCs with magnetic-activated cell sorting-purified allogenic T cells (1 × 10^5/well) for 6 days in 1:20 DC:T cell ratio. The summarized results are shown as mean % ± SD of three experiments with different DCs donors. *p < 0.05, **p < 0.01, ***p < 0.001 compared to control untreated DCs (other comparisons are indicated by lines in Figure 5).

Cytokines	Treatment	Expression of cytokines in $CD4^+$ T cells	Mean ± SD
IL-4 (Expression shown as %)	Control	12.4<br>6.9<br>8.1	9.81 ± 2.89
	LPS/IFN-γ	14.0<br>7.16<br>9.2	10.1 ± 3.51
	ES L1	24.8<br>17.9<br>21.4	21.4 ± 3.45***
	TsGal-1-like	19.2<br>12.2<br>17.2	16.2 ± 3.61**
	TsGal-1-like/Lac	16.2<br>6.2<br>15.5	12.6 ± 5.58
	Lac	14.2<br>4.8<br>6.2	8.4 ± 5.07
IL-10 (Expression shown as %)	Control	14.9<br>7.14<br>8.3	10.1 ± 4.19
	LPS/IFN-γ	15.4<br>7.13<br>9.4	10.6 ± 4.27
	ES L1	22.0<br>15.6<br>20.5	19.4 ± 3.35***
	TsGal-1-like	21.4<br>16.8<br>19.5	19.2 ± 2.31***
	TsGal-1-like/Lac	16.7<br>12.6<br>16.6	15.3 ± 2.34*
	Lac	15.0<br>8.4<br>9.1	10.8 ± 3.65
IFN-γ (Expression shown as %)	Control	4.16<br>4.91<br>3.9	4.3 ± 0.52
	LPS/IFN-γ	11.5<br>12.7<br>12.2	12.1 ± 0.60***
	ES L1	5.79<br>4.29<br>4.8	5.0 ± 0.76
	TsGal-1-like	5.56<br>3.25	4.4 ± 1.16

EXCLI Journal 2022;21:793-813 – ISSN 1611-2156
Received: April 14, 2022, accepted: May 31, 2022, published: June 03, 2022

		4.5	
	TsGal-1-like/Lac	6.0<br>4.77<br>5.0	5.3 ± 0.65
	Lac	5.4<br>3.5<br>4.1	4.3 ± 0.97

EXCLI Journal 2022;21:793-813 – ISSN 1611-2156
Received: April 14, 2022, accepted: May 31, 2022, published: June 03, 2022

Raw data concerning **Figure 6. Tolerogenic properties and functions of TsGal-1-like proteins-treated DCs** - the expression of Foxp3 in $CD4^+CD25^+$ T cell population, as well as IL-10 and TGF-β within the same T cell population, analyzed by flow cytometry, is presented as percentage (%) for each of three independent experiments, all upon the co-culture of treated and non-treated DCs with magnetic-activated cell sorting-purified allogenic T cells (Tly) (1 × 10^5/well) for 3 days in 1:50 DC:T cell ratio and then re-stimulated with interleukin (IL)-2 (2 ng/ml) for another 3 days. The summarized results for the expression of $CD4^+CD25^+Foxp3^+$ T regulatory cells and IL-10 and TGF-β within $CD4^+CD25^+$ T cells are shown as the mean % ± SD from three different experiments. **p < 0.01, ***p < 0.005 compared to control untreated DCs (other comparisons are indicated by lines in Figure 6).

**Transcriptional regulator or cytokine**	**Treatment**	**Expression of proteins in $CD4^+$CD25+ T cells**	**Mean ± SD**
Foxp3 (Expression shown as %)	Control	3.24<br>4.5<br>4.8	4.2 ± 0.83
	LPS/IFN-γ	3.38<br>5.5<br>5.1	4.7 ± 1.13
	ES L1	15.8<br>11.2<br>11.8	12.9 ± 2.50***
	TsGal-1-like	11.6<br>13.0<br>10.8	11.8 ± 1.11***
	TsGal-1-like/Lac	10.8<br>9.6<br>8.8	9.7 ± 1.01***
	Lac	5.9<br>4.9<br>5.5	5.4 ± 0.50
IL-10 (Expression shown as %)	Control	11.0<br>7.2<br>10.5	9.6 ± 2.06
	LPS/IFN-γ	15.5<br>14.0<br>16.8	15.4 ± 1.40
	ES L1	18.5<br>27.5<br>25.5	23.8 ± 4.73***
	TsGal-1-like	25.8<br>29.3<br>23.7	26.3 ± 2.83***
	TsGal-1-like/Lac	22.1<br>19.1<br>18.1	19.8 ± 2.08**
	Lac	3.8<br>17.1<br>15.5	12.1 ± 7.26
TGF-β (Expression shown as %)	Control	35.9<br>42.4<br>33.6	37.3 ± 4.56
	LPS/IFN-γ	40.1<br>48.7<br>42.2	43.7 ± 4.48
		70.7	

EXCLI Journal 2022;21:793-813 – ISSN 1611-2156
Received: April 14, 2022, accepted: May 31, 2022, published: June 03, 2022

	ES L1	73<br>72.1	71.9 ± 1.19***
	TsGal-1-like	80<br>74<br>70.1	74.7 ± 4.99***
	TsGal-1-like/Lac	67.8<br>63.6<br>64.4	65.3 ± 2.23**
	Lac	50.1<br>55.4<br>48.2	51.2 ± 3.73
